# Health-related quality of life and symptom-specific functional impairment among patients treated with parenterally administered complement inhibitors for paroxysmal nocturnal hemoglobinuria

**DOI:** 10.1007/s00277-024-06088-x

**Published:** 2024-11-18

**Authors:** David Dingli, Avery A. Rizio, Lynne Broderick, Kaitlin LaGasse, Sloan Rucker, Michelle K. Carty, Elise Burton, Shaquilla Gordon, Glorian P. Yen, Jincy Paulose, Anumaxine Geevarghese, Soyon Lee

**Affiliations:** 1 Mayo Clinic, Rochester, MN USA; 2 QualityMetric, An IQVIA Business, 1301 Atwood Avenue, Suite 216E, Johnston, RI 02919 USA; 3 Jackson Heights, New York, NY USA; 4 Altantic Beach, Florida, FL USA; 5 Novartis Pharmaceuticals Corporation, East Hanover, NJ USA

**Keywords:** PNH, Patient-reported outcomes, Survey, Qualitative, Interview, Fatigue

## Abstract

This study describes the health-related quality of life (HRQoL) and symptom-specific functional impairment of patients with paroxysmal nocturnal hemoglobinuria (PNH) in a real-world setting. US-based adults with PNH treated with a parenterally administered complement inhibitor (PACI) for ≥ 6 months completed an online, cross-sectional, observational survey; a subset of patients also participated in semi-structured qualitative interviews. The survey included the PROMIS^®^ 29 + 2 Profile v2.1 (PROMIS 29 + 2) to measure HRQoL. The FACIT-Fatigue, Neuro-QOL Item Bank v2.0 Cognitive Function Short Form, and PROMIS Item Bank v1.0 Dyspnea Functional Limitations 10a Short Form measured symptom-specific functional impairment. For each patient with PNH who completed the online survey, 3 age- and sex-matched adults from the general population (GP) also completed the survey. The HRQoL and functional impairment of the PNH sample were compared to that of the GP sample. The association between HRQoL/functional impairment and fatigue severity for the PNH sample was also investigated. Compared to the age- and sex-matched GP sample, patients treated with PACIs for PNH had significantly worse HRQoL and greater functional impairment for all measured domains (*p* < 0.05). Within the PNH sample, statistically significant associations (*p* < 0.05) were observed between fatigue severity and HRQoL/functional impairment for all outcomes except the PROMIS 29 + 2 Sleep Disturbance domain. Interview participants described fatigue-related impairments in their physical, social, and cognitive functioning. Despite receiving treatment for PNH, patients experienced deficits in HRQoL and functional impairment, suggesting that opportunities to improve patient-relevant outcomes through treatment should be identified.

## Introduction

Paroxysmal nocturnal hemoglobinuria (PNH) is a rare, acquired, life-threatening, clonal hematopoietic stem cell disorder with a prevalence in the United States (US) estimated at 12 to 13 per million individuals and an incidence rate of 6 cases per 1 million person-years between 2016 and 2017 [1–3]. PNH is caused by somatic mutations in the *PIGA* gene; this mutation causes red blood cells to be deficient in GPI-anchored proteins including complement regulators CD55 and CD59, rendering the red blood cells vulnerable to complement-mediated intravascular and extravascular hemolysis [1, 2]. In addition to hemolysis, PNH is associated with a high risk of thrombosis in unusual areas such as hepatic or cerebral veins; PNH may also be associated with bone morrow failure and renal failure [1, 2, 4, 5]. Patients with PNH experience a myriad of symptoms including fatigue, the most commonly reported symptom, headache, dyspnea, abdominal pain, erectile dysfunction, and gastrointestinal spasms, with approximately 93% of patients experiencing at least 1 symptom [6–8]. Some of these symptoms, including dyspnea, abdominal pain, erectile dysfunction, and gastrointestinal spasms, result from a drop in nitric oxide – which is scavenged by free hemoglobin – leading to smooth muscle contraction and vasoconstriction [9].

Multiple treatment options exist for patients with PNH. Allogeneic stem cell transplant is a curative option for patients with PNH, although it is not recommended for many patients due to the associated risks and the increasing availability of less toxic therapeutic options that prolong survival without the morbidity associated with transplant [10]. Stem cell transplant may be considered for patients with severe aplastic anemia, for patients who do not respond to complement therapy, or when patients continue to experience cytopenias or thrombotic events [11, 12]. The best candidates for stem cell transplant include younger patients and those with a fully matched sibling donor [11]. The US Food and Drug Administration (FDA) has approved 4 parenterally administered complement inhibitors to treat PNH: eculizumab, ravulizumab, and crovalimab are C5 inhibitors that block distal complement activation to prevent intravascular hemolysis [13–15], while pegcetacoplan is a C3 inhibitor that blocks proximal complement activation to reduce intravascular and extravascular hemolysis [16]. The FDA has also recently approved iptacopan, an orally administered proximal complement factor B inhibitor that targets the alternative complement pathway [17], and danicopan, a factor D inhibitor for add-on therapy to ravulizumab or eculizumab [18].

Published data from clinical trials have demonstrated that treatment with parenterally administered complement inhibitors is associated with improvement in patient-relevant experiences. For example, patients in a phase 3 trial who were treated with eculizumab experienced significantly greater reductions in fatigue, pain, dyspnea, loss of appetite, and insomnia than patients on placebo [19]. Significantly greater improvements in patient-reported global health status and role, social, cognitive, physical, and emotional functioning were also observed relative to placebo. A phase 3, open-label trial demonstrated that ravulizumab was non-inferior to eculizumab in reducing fatigue and improving health-related quality of life (HRQoL) [20]. Similarly, a phase 3, open-label trial demonstrated that 73% of patients treated with pegcetacoplan experienced a clinically significant improvement in fatigue [21]; improvements in patient-reported global health status and HRQoL were also observed [22].

While treatment-related improvements in symptom-specific functional impairment and HRQoL have been documented, particularly in the context of clinical trials, the possibility remains that – despite treatment – patients still experience burden due to their PNH. Quantifying deficits in HRQoL can be accomplished by comparing health status between patients with PNH and individuals from the general population without PNH. This approach has been taken in the past, but findings are mixed. For example, patients enrolled in 2 clinical trials of ravulizumab and eculizumab were compared to a multi-national sample of patients from the general population; analyses indicated that treated patients with PNH had better physical, emotional, and cognitive functioning than the general population sample, and lower levels of nausea/vomiting, pain, insomnia, appetite loss, and constipation/diarrhea. Patients on ravulizumab also reported better global health status and less fatigue than the general population sample [23]. In contrast, 2 other studies of patients recruited through advocacy groups who were being treated with eculizumab or ravulizumab reported greater fatigue and worse HRQoL as compared to pre-existing reference values from the general population [8, 24]. Taken together, existing literature presents an inconsistent picture of the disease experience among patients treated for PNH. Studies that report deficits have typically used general population scores from pre-existing samples, where participant characteristics such as age could differ notably from the PNH sample group, possibly contributing to the observed differences. Alternatively, the carefully controlled clinical trial setting could have promoted substantial increases in health status that may not be observed in real-world settings.

Considering the current state of the literature, additional research is needed to explore the degree to which patients with PNH may experience unresolved symptoms and subsequent deficits in HRQoL despite treatment with parenterally administered complement inhibitors. Studies exploring HRQoL and patient experience among treated patients are warranted to help inform treatment strategies. The overall objective of the current study was to describe the HRQoL and symptom-specific functional impairment of patients being treated for PNH in a real-world setting. This objective was met by administering an online survey to compare the HRQoL and symptom-specific functional impairment of patients treated with a parenterally administered complement inhibitor to a sample of age- and sex-matched participants from the general population and then using qualitative concept elicitation interviews to further explore and contextualize these experiences. Both survey data and qualitative interviews were also used to more specifically evaluate patients’ fatigue experiences, particularly in terms of its association with HRQoL.

## Methods

### Study sample and procedures

This real-world, mixed-methods, observational study included a cross-sectional, online survey administered to a sample of patients with PNH and a sample of age- and sex-matched participants from the general population, in addition to qualitative interviews conducted among a subset of the PNH patient sample. Participants from the general population were not invited to complete interviews. Prior to data collection, the online survey and the qualitative interview guide were reviewed by a panel of 3 patients with PNH; revisions to the materials were made based on their recommendations regarding relevant content and patient-appropriate language for medical or disease-related terminology. The study protocol and associated study materials were approved by an independent Institutional Review Board (Western Institutional Review Board-Copernicus Group [WCG]). All participants provided informed consent and were provided an honorarium for their time completing the study procedures. All data collected through the study were provided by participant self-report.

#### PNH patient sample

A convenience sample of patients with PNH who were being treated with a parenterally administered complement inhibitor was recruited through existing databases of potential participants and healthcare providers willing to distribute messages about the study, social media engagement, and collaboration with the Aplastic Anemia and MDS International Foundation. Potential participants were screened over the phone for eligibility. Patients with PNH were eligible to participate if they were at least 18 years old, currently resided in the US, were fluent in English, were currently treated with eculizumab, ravulizumab, or pegcetacoplan and had been on that treatment for at least 6 months, and were able to provide confirmation of their PNH diagnosis. Patients were ineligible to participate if they had taken part in a clinical trial in the past 6 months. All eligible patients were invited to complete an online survey, which was fielded from March – September 2023. At the time of the survey, crovalimab, iptacopan, and danicopan were not commercially available.

A subset of patients with PNH also completed an hour-long concept elicitation interview via online conferencing software. All patients with PNH who completed the online survey were invited to take part in an interview, until 25 interviews were completed. Interviews took place 1–3 weeks after survey completion, such that patients who had taken the survey more than 3 weeks prior were no longer eligible to complete an interview. This timeframe was selected to achieve an optimal balance between accommodating participants’ schedules, making every reasonable attempt to ensure those who wanted to participate could, and ensuring that interviews were held soon enough after the survey was completed so that participants could easily discuss their survey responses.

#### General population sample

For every 1 patient with PNH who completed the online survey, 3 adults from the US general population who did not have PNH were recruited to participate. The general population participants were recruited through pre-existing databases and were screened over the phone for eligibility. Potential participants from the general population were considered eligible if they were at least 18 years old, currently resided in the US, were fluent in English, and did not have a diagnosis of PNH. Each general population participant was matched on age (+/- 3 years) and sex to a patient from the PNH sample.

### Online survey

#### PNH patient sample

Relevant demographic criteria, clinical characteristics, and treatment experiences necessary to confirm eligibility were collected at screening. Additional information such as educational attainment, time since diagnosis, and most recent hemoglobin level were collected through the online survey.

HRQoL was measured by the PROMIS^®^ 29 + 2 Profile v2.1 (PROMIS Profile), which is intended to assess well-being of adults across different types of diseases or patient populations. The PROMIS Profile includes 9 scales: physical function, anxiety, depression, fatigue, sleep disturbance, ability to participate in social roles and activities, pain interference, cognitive functioning, and pain intensity.

The Functional Assessment of Chronic Illness Therapy – Fatigue Scale (FACIT-Fatigue), Neuro-QOL Item Bank v2.0 Cognitive Function Short Form (NeuroQOL Cognitive Function SF), and PROMIS^®^ Item Bank v1.0 Dyspnea Functional Limitations 10a Short Form (PROMIS Dyspnea Limitations SF) were administered as measures of symptom-specific functional impairment, targeting impairment due to known symptoms of PNH. While the PROMIS Profile includes items to evaluate fatigue and cognitive functioning, the additional surveys were administered to provide a more detailed evaluation of these patient-relevant concepts.

These instruments were selected for inclusion in the study because they address concepts that are relevant to patients with PNH, are relevant to individuals from the general population who may or may not be ill, and have well-established scoring systems.

#### General population sample

Age and sex were collected at screening; additional demographic characteristics such as educational attainment were collected through the online survey. Participants from the general population completed the PROMIS Profile, FACIT-Fatigue, NeuroQOL Cognitive Function SF, and PROMIS Dyspnea Limitations SF.

### Qualitative interviews

Interviews followed a semi-structured interview guide developed specifically for this study. Prior to each interview, the interviewer received a summary of the participant’s survey data detailing the symptoms they reported and scores on the measures of HRQoL and symptom-specific functional impairment described above. The interviewer reviewed this summary and prepared for each interview so that targeted questions could be asked. The interview guide was structured such that participants were first asked to describe the symptoms they reported in the online survey, specifically the frequency, severity, and timing of breakthrough symptoms, and then were asked to similarly describe any other PNH-related symptoms they experienced. Participants were next asked about HRQoL domains for which their scores indicated greater than average impairment and were asked to describe any additional ways in which PNH has affected their lives that might not have been captured in the online survey.

### Analyses

Both quantitative and qualitative analyses were conducted using all available survey and interview data, respectively. Quantitative analyses were conducted using SAS version 9.4.

Qualitative analyses were conducted using NVivo. All transcripts were reviewed by the study team to ensure accuracy and completeness. The first 10% of transcripts were reviewed alongside the interview audio recordings to ensure the transcription quality met the study team’s standards. The remaining transcripts were reviewed using the audio recordings as needed to try to fill in any inaudible gaps.

To ensure high quality coding, 2 members of the study team independently coded the same first 20% of transcripts, comparing coding to assess reliability. Once any coding discrepancies were addressed and consensus was reached through internal discussions, the remaining transcripts were divided into groups, and each group was coded by a single study team member. The qualitative study lead reviewed all coding prior to analysis.

#### Sample characteristics

For both the PNH and general population samples, descriptive statistics were used to describe demographic characteristics. Descriptive statistics were also used to describe disease characteristics and treatment experiences for the PNH sample.

#### Describing HRQoL and symptom-specific functional impairment

To help describe the HRQoL and symptom-specific functional impairment of patients with PNH, analyses using data from the online survey were conducted to compare the HRQoL and symptom-specific functional impairment of patients with PNH to the general population sample. Mann-Whitney tests were used to compare scores on the PROMIS Profile, FACIT-Fatigue, NeuroQOL Cognitive Function SF, and PROMIS Dyspnea Limitations SF between the 2 samples. P-values were adjusted for multiple comparisons using the Benjamini-Hochberg correction; statistical significance was assessed at a corrected alpha of 0.05.

Concept elicitation data were analyzed to further contextualize the HRQoL and symptom-specific functional impairment experienced by patients with PNH. Transcript data were coded in NVivo using thematic analysis to identify patterns in patient responses concerning HRQoL and functional impairment.

#### Association between fatigue severity and HRQoL/symptom-specific functional impairment among patients with PNH

Quantitative analyses of survey data were conducted to explore the association between fatigue severity and HRQoL/symptom-specific functional impairment among patients with PNH. Patients with PNH were stratified according to the severity of their fatigue, as evaluated over the past 7 days (aligning with the recall period of the other instruments administered during the study): very mild or mild vs. moderate, severe, or very severe. No patients reported no fatigue. Differences in HRQoL and symptom-specific functional impairment scores between these 2 groups of patients were evaluated using independent samples t-tests or Mann-Whitney tests. P-values were adjusted for multiple comparisons using the Benjamini-Hochberg correction; statistical significance was assessed at a corrected alpha of 0.05.

Matrix coding queries of qualitative interview data were conducted to examine the intersection of HRQoL impacts by symptoms.

## Results

In keeping with the objectives of the mixed-methods study, results from the survey and qualitative interviews are integrated when data from one method provide additional context (e.g., qualitative descriptions of impacts on physical functioning related to fatigue add to results from the online survey), or aid in the interpretation of data collected from the other method (e.g., qualitative descriptions of the unpredictability of PNH symptoms shed light on social functioning impacts reported in the online survey).

### Analytic sample

**Fig. 1:**
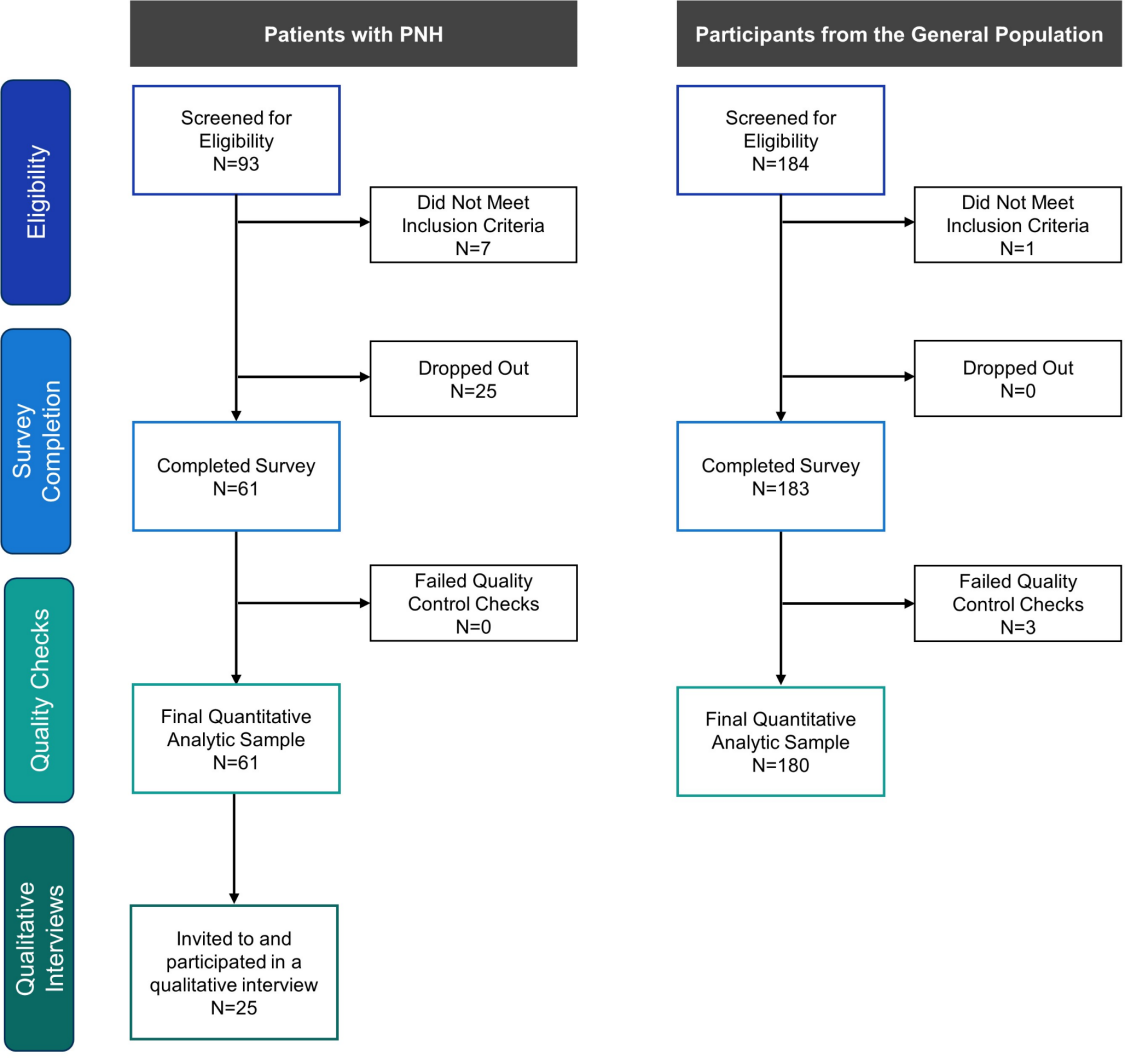
Flowchart of participant disposition. *Abbreviation* PNH, paroxysmal nocturnal hemoglobinuria

Sixty-one patients with PNH completed the online survey; all passed quality control checks and thus were included in the quantitative analyses. Of these patients, 25 were invited to and completed, a 1-on-1 qualitative interview (Fig. 1).

For every patient with PNH who completed the survey, 3 participants from the general population also completed the survey. Of these 183 participants, 3 failed quality control checks and thus were excluded from the analytic sample. These 3 participants were in the fastest 10% of survey completers and provided the same response to all FACIT-Fatigue items, despite some items being worded in the opposite direction. The final general population analytic sample included 180 participants (Fig. 1).

### Sample characteristics

Participant demographic characteristics are provided in Table 1. Reflecting the matching procedure, the distribution of female and male general population participants (72.2% and 27.8%, respectively) matched that of the PNH survey sample (72.1%, and 27.9%, respectively). While general population participants were matched +/- 3 years to the patient sample, the average age of the 2 groups ultimately differed by less than half a year (mean age = 47.3 for the general population sample and 46.9 for the PNH survey sample). The subset of patients with PNH who participated in the qualitative interviews generally reflected the broader survey sample in terms of mean age (46.0 years old), sex (72.0% female), gender identity (68.0% female), and race/ethnicity (84.0% Caucasian or White). More racial diversity was observed for the general population sample than for the PNH patient sample.

**Table 1:** Participant demographic characteristics

	Participants from the general population (*N* = 180)		Patients with PNH who completed the survey (*N* = 61)		Patients with PNH who also completed an interview (*N* = 25)	
**Continuous Variables**	**Mean (SD)**		**Mean (SD)**		**Mean (SD)**	
Age (years)	47.3 (14.1)		46.9 (14.3)		46.0 (14.0)	
**Categorical Variables**	**n**	**%**	**n**	**%**	**n**	**%**
**Sex assigned at birth**						
Female	130	72.2	44	72.1	18	72.0
Male	50	27.8	17	27.9	7	28.0
**Gender identity**						
Female	127	70.6	43	70.5	17	68.0
Male	49	27.2	17	27.9	7	28.0
Nonbinary or Gender non-conforming	2	1.1	1	1.6	1	4.0
I do not wish to answer	2	1.1	0	0.0	0	0.0
**Race and/or ethnicity** ^**a**^						
Caucasian or White	80	44.4	47	77.0	21	84.0
Black	28	15.6	7	11.5	3	12.0
Hispanic or Latino	20	11.1	6	9.8	2	8.0
Asian	49	27.2	3	4.9	1	4.0
American Indian or Alaska Native	2	1.1	1	1.6	1	4.0
Native Hawaiian or Other Pacific Islander	4	2.2	1	1.6	1	4.0
Other	4	2.2	2	3.3	0	0.0
I do not wish to answer	4	2.2	3	4.9	0	0.0
**Highest grade level completed**						
4-year college degree (e.g., BA, BS)	55	30.6	19	31.1	9	36.0
Graduate or professional degree (e.g., MBA, MS, MD, PhD)	31	17.2	11	18.0	4	16.0
Associate degree (2-year college degree)	27	15.0	10	16.4	2	8.0
Some college but no degree	24	13.3	8	13.1	3	12.0
High school or equivalent (e.g., GED)	14	7.8	6	9.8	4	16.0
Some graduate but no degree	13	7.2	4	6.6	2	8.0
Technical school	14	7.8	2	3.3	0	0.0
Some high school	2	1.1	1	1.6	1	4.0
**Employment status** ^**a**^						
Employed full-time	82	45.6	20	32.8	13	52.0
Employed part-time	35	19.4	13	21.3	5	20.0
Retired	15	8.3	13	21.3	4	16.0
Self-employed	13	7.2	6	9.8	1	4.0
Homemaker	8	4.4	5	8.2	2	8.0
Disabled/Unable to work	13	7.2	5	8.2	2	8.0
Unemployed and not looking for work	5	2.8	5	8.2	1	4.0
Student	3	1.7	0	0.0	0	0.0
Unemployed but looking for work	9	5.0	0	0.0	0	0.0

^a^ More than one response allowed per participant; percentage may sum to > 100%

*Note* Responses are ordered according to frequency of the PNH survey sample except for responses of “I do not wish to respond,” which are presented last

*Abbreviations* PNH, paroxysmal nocturnal hemoglobinuria; SD, standard deviation

Select clinical characteristics of the PNH patient sample are provided in Table 2. On average, patients had been diagnosed with PNH 11 years prior and had been on their current complement inhibitor for approximately 3 years. 60.7% of the PNH survey sample were currently treated with ravulizumab, while the remainder were being treated with pegcetacoplan (21.3%) or eculizumab (18.0%). About half of the interview participants (*n* = 13; 52.0%) were being treated with ravulizumab at the time of the interview, with a mean treatment time of 2.7 years. Eight interview participants (32.0%) were being treated with pegcetacoplan, with a mean treatment time of 1.7 years, and 4 interview participants (16.0%) were being treated with eculizumab, with a mean treatment time of 10.6 years. Altogether, patients had been on their current complement inhibitor for an average of 3.7 years.

**Table 2:** Clinical characteristics and treatment experiences of patients with PNH

	Patients who completed the survey (*N* = 61)		Patients who also completed an interview (*N* = 25)	
**Continuous Variables**	**Mean (SD)**		**Mean (SD)**	
Time since PNH diagnosis, years	11.0 (9.3)		10.6 (8.3)	
Time since start of treatment with current complement inhibitor, years	3.2 (2.9)		3.7 (3.7)	
Time since start of treatment with first complement inhibitor, years^a^	9.5 (5.8)		9.1 (5.2)	
Time since last infusion, weeks	2.9 (2.7)		2.2 (2.8)	
Most recent hemoglobin level (gm/dL)^b^	10.8 (1.8)		11.1 (2.2)	
Time since most recent hemoglobin test, days^b^	27.6 (37.5)		20.8 (36.1)	
**Categorical Variables**	**n**	**%**	**n**	**%**
**Current treatment**				
Ravulizumab (Ultomiris)	37	60.7	13	52.0
Pegcetacoplan (Empaveli)	13	21.3	8	32.0
Eculizumab (Soliris)	11	18.0	4	16.0
**Diagnosis of bone marrow disorder**				
I have not been diagnosed with any of these conditions	39	63.9	14	56.0
Aplastic anemia (AA) or severe aplastic anemia (SAA)	18	29.5	9	36.0
Myelodysplastic syndrome (MDS)	0	0.0	0	0.0
Acute myeloid leukemia (AML)	0	0.0	0	0.0
Other blood disorder/failure of bone marrow	1	1.6	0	0.0
I do not know	3	4.9	2	8.0

^a^ Reported for patients who responded “yes” to having received treatment with a different complement inhibitor prior to beginning their current complement inhibitor (*n* = 29 for survey sample and 14 for interview sample)

^b^ Most recent hemoglobin was provided by patient self-report; 8 survey sample patients and 2 interview patients reported they did not know their hemoglobin levels

*Note* Responses are ordered according to frequency, except for ordinal variables, which retain the order of responses presented in the survey

*Abbreviations* PNH, paroxysmal nocturnal hemoglobinuria; SD, standard deviation

### HRQoL and symptom-specific functional impairment

**Fig. 2:**
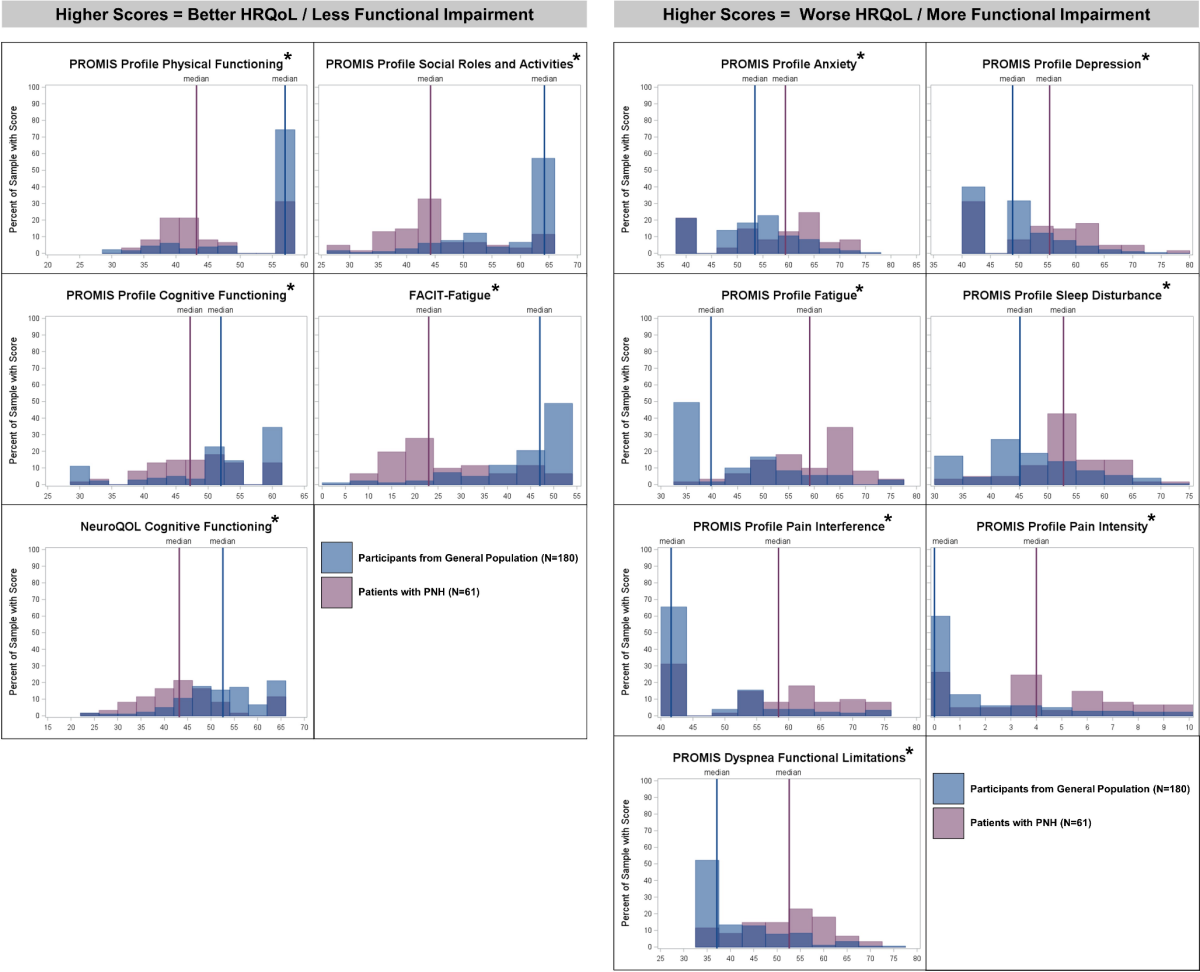
Distribution of HRQoL and symptom-specific functional impairment scores between patients with PNH and age- and sex-matched participants from the general population. *Notes* x-axis corresponds to scores for each domain or measure, restricted to the approximate possible score range. * = statistically significant difference, as evaluated by Mann-Whitney tests. *Abbreviations* FACIT, Functional Assessment of Chronic Illness Therapy; HRQoL, health-related quality of life; PNH, paroxysmal nocturnal hemoglobinuria; PROMIS, Patient-Reported Outcomes Measurement Information System; QOL, quality of life

Patients with PNH had worse HRQoL and greater symptom-specific functional impairment than the general population sample (Table 3; Fig. 2). Statistically significant differences between the 2 samples were observed for all scales of the PROMIS Profile, as well as for the FACIT-Fatigue, NeuroQOL Cognitive Function SF, and PROMIS Dyspnea Limitations SF (corrected *p* < 0.05 for all).

**Table 3:** Comparison of HRQoL and symptom-specific functional impairment between patients with PNH and age- and sex-matched participants from the general population

	Disease status							
	Patients with PNH			Participants from the general population				
Instrument	*n*	Mean (SD)	Median (IQR)	*n*	Mean (SD)	Median (IQR)	*P*	*P*_BH_
**PROMIS** ^®^ **29 + 2 Profile v2.1**								
Physical functioning ^↑^	61	45.99 (8.16)	43.20 (39.80, 57.00)	180	52.65 (7.97)	57.00 (48.60, 57.00)	< 0.001	< 0.001
Anxiety	61	56.65 (10.48)	59.40 (51.40, 65.20)	180	52.63 (8.42)	53.45 (48.10, 57.50)	0.002	0.002
Depression	61	53.45 (10.03)	55.40 (41.00, 60.50)	180	48.64 (7.56)	48.90 (41.00, 52.55)	< 0.001	< 0.001
Fatigue	61	58.68 (9.04)	59.10 (51.00, 64.70)	180	43.61 (11.64)	39.80 (33.70, 49.85)	< 0.001	< 0.001
Sleep disturbance	61	52.63 (7.94)	52.80 (51.00, 56.10)	180	46.43 (9.82)	45.10 (41.20, 52.75)	< 0.001	< 0.001
Ability to participate in social roles and activities ^↑^	61	45.45 (9.41)	44.20 (40.20, 50.20)	180	57.70 (9.01)	64.20 (51.80, 64.20)	< 0.001	< 0.001
Pain interference	61	56.16 (11.36)	58.40 (41.60, 64.40)	180	47.56 (9.23)	41.60 (41.60, 55.70)	< 0.001	< 0.001
Cognitive functioning ^↑^	61	47.76 (7.83)	47.20 (41.00, 53.70)	180	50.94 (10.38)	52.00 (46.75, 61.20)	0.002	0.002
Pain intensity	61	3.74 (3.02)	4.00 (0.00, 6.00)	180	1.47 (2.47)	0.00 (0.00, 2.00)	< 0.001	< 0.001
**FACIT-Fatigue**								
Total score ^↑^	61	26.41 (12.29)	23.00 (17.00, 35.00)	180	42.78 (11.36)	47.00 (39.00, 51.00)	< 0.001	< 0.001
**Neuro-QOL Item Bank v2.0 – Cognitive Function – SF**								
Total score ^↑^	61	44.51 (9.63)	43.20 (38.00, 49.10)	180	52.27 (9.17)	52.55 (46.90, 59.10)	< 0.001	< 0.001
**PROMIS Item Bank v1.0 – Dyspnea Functional Limitations – SF 10a**								
Total score	61	51.15 (9.28)	52.60 (45.00, 59.00)	180	41.58 (8.91)	37.10 (34.80, 46.05)	< 0.001	< 0.001

^↑^ Indicates that higher scores are better; for all other domains, higher scores are worse

*Note* All differences evaluated with Mann-Whitney test for non-normally distributed variables

*Abbreviations* BH, Benjamini-Hochberg (correction for multiple comparisons); FACIT, Functional Assessment of Chronic Illness Therapy; HRQoL, health-related quality of life; IQR, interquartile range; PNH, paroxysmal nocturnal hemoglobinuria; PROMIS, Patient-Reported Outcomes Measurement Information System; QOL, quality of life; SD, standard deviation; SF, short form

Interview participants provided insights into the ways PNH impacted their HRQoL and reported experiencing impairments across the following domains: emotional functioning, physical functioning, employment/career and financial well-being, social functioning, and cognitive functioning (see Table 4 for example quotes). As described below, participants attributed some of their functional limitations, including those related to physical and social functioning, to specific PNH symptoms such as dyspnea and fatigue.

Almost all interview participants reported impacts on emotional functioning (*n* = 23; 92.0%). When asked to further describe their responses to survey items regarding emotional functioning, participants described experiencing feelings of anxiety which they linked directly to having PNH and the unpredictability of PNH symptoms. During interviews, patients also discussed the overall emotional strain associated with having PNH, as well as feelings of worry, frustration, and stress, “[T]here’s always the—that lingering… worry that you know, if I expire earlier than, than anticipated, you know, will my—will my family be okay?” (ID 0001, male, age 37).

88% (*n* = 22) of interview participants described the ways PNH impacted their physical functioning, most of which were attributed directly to PNH-related fatigue (described below). Five participants (out of 18 who reported dyspnea; 27.8%) also linked physical functioning impairments to dyspnea and described avoiding situations where physical activities like going for a short walk, climbing stairs, or walking uphill might exacerbate it, “[G]oing up the stairs can really get me out of breath at times… there are times where [my husband] has to help me walk up the stairs” (ID 0005, female, age 28).

Impacts on the ability to work full- or part-time, either currently or in the past. as well as general career impacts, were also reported by interview participants (*n* = 18; 72.0%). They described missing half or full days of work due to their PNH symptoms and/or due to receiving infusions. Impacts not captured by the online survey, including an altered career trajectory due to PNH and decreased productivity while at work due to cognitive symptoms such as brain fog, also emerged during interviews. Approximately one-third of participants (*n* = 9; 36.0%) further reported experiencing financial difficulties related to their inability to work full-time and the costs of health insurance, medical care, and PNH treatments.

Aligning with the survey data on social functioning, interview participants (*n* = 18, 72.0%) described difficulties in maintaining social relationships and activities, including romantic relationships, attributing many of these difficulties to fatigue (described below). During interviews, participants elaborated on how the unpredictable nature and severity of PNH symptoms impacted their ability to even make plans, “[I]t’s hard to kind of like plan life around PNH… because of the fact that you just aren’t totally sure what it’s going to be… you kind of have to re-evaluate what takes priority, because sometimes you will have to kind of drop something…” (ID 0033, female, age 29).

Interview participant accounts of cognitive functioning impacts (*n* = 14; 56.0%) reflected what was captured in the survey, including slower cognitive processing and brain fog, and allowed for an in-depth discussion on what these impacts look like in everyday functioning, including difficulties with word retrieval, “I’ll say a word and it will be close to the word I wanted to say, but it’ll be a total other word… there was a horse drawn carriage and I says, ‘Oh, cabbage rides’” (ID 0002, female, age 64).

Similarly, reports of sleep disturbance (*n* = 9; 36.0%) aligned with survey results as participants reported difficulty falling and staying asleep (*n* = 9; 36.0%) due to PNH symptoms such as pain, difficulty breathing, heart palpitations, or feelings of anxiety related to PNH.

**Table 4:** Example patient quotes describing HRQoL impacts due to PNH

HRQoL Domain	Example Quotes
Emotional functioning	I think it just makes you… I want to say—sometimes like grumpier… it just affects your mental health that you know… because you’re always dealing with it… it’s not as easy to overlook things or to—you know, let things just go by. *(ID 0015*, *female*, *age 60)*
Physical functioning	…that fatigue, it—it—it encompasses everything…you know, even—even chores and everything…I’ve got to pace myself…I can’t go food shopping in the morning and then start cleaning the house in the afternoon. *(ID 0018*, *female*, *age 75)*
Work/career-related impacts	I’m able to work but as I said, am I at my 100% best?…[But] say if I want to jump the ship to a different employer for a better salary or—er—better role, I’m afraid whether I can er, fulfil the demands of that role because of my [treatment] commitments. Every eight weeks I need to do this, do that, so will—will my new boss be as sympathizing as the current one is or whatnot. So, those kind of things, while it doesn’t directly impact it, but it has impacted [my career growth] 200%. *(ID 0024*, *male*, *age 45)*
Social functioning	If I’m fatigued and tired, it’s hard for me to get up to even go for one, [laughs] and then getting there— I’m already ready to go home, just because I feel tired and just out of place I guess. I don’t feel like I can completely function with everyone else. (*ID 0009*, *female*, *age 33)*
Cognitive functioning	[S]ome written instructions… I have to read them like five or six times, because I just can’t focus or whatever tasks I have to do on the computer… I start thinking of something else… it’s like I’m reading it, but I’m not really grasping what I’m reading. (*ID 0004*,* female*, *age 46)*
Sleep disturbance	[G]etting back in bed for some reason, it can be really difficult for me, like rolling around getting my pillow right, getting the blankets—like, I get out of breath and then I just lay there… in the past, sometimes when my numbers were super low, and I was trying to get comfortable, I would get so frustrated, I would start crying ‘cause I would be, like, out of breath and my heart racing… *(ID 0011*, *female*, *age 37)*
Financial well-being	I would love to have a full-time career right now…I feel like I’m not reaching my full potential income-wise, because of PNH…I would love to be making a lot more money than I am right now and it’s just difficult to commit to something with the brain fog and the fatigue. It’s a big—it’s a heavy, heavy burden…it’s very frustrating to, to navigate. Um—luckily right now, I’m working for family, so they are very understanding, but in that sense, I’m not—I feel reaching my potential, income-wise because of that. So, to me, it’s the most bothersome and—and absolutely the most frustrating in my eyes. *(ID 005*, *female*, *age 28)*

*Abbreviation* PNH, paroxysmal nocturnal hemoglobinuria

### Association between fatigue severity and HRQoL/symptom-specific functional impairment among patients with PNH

**Fig. 3:**
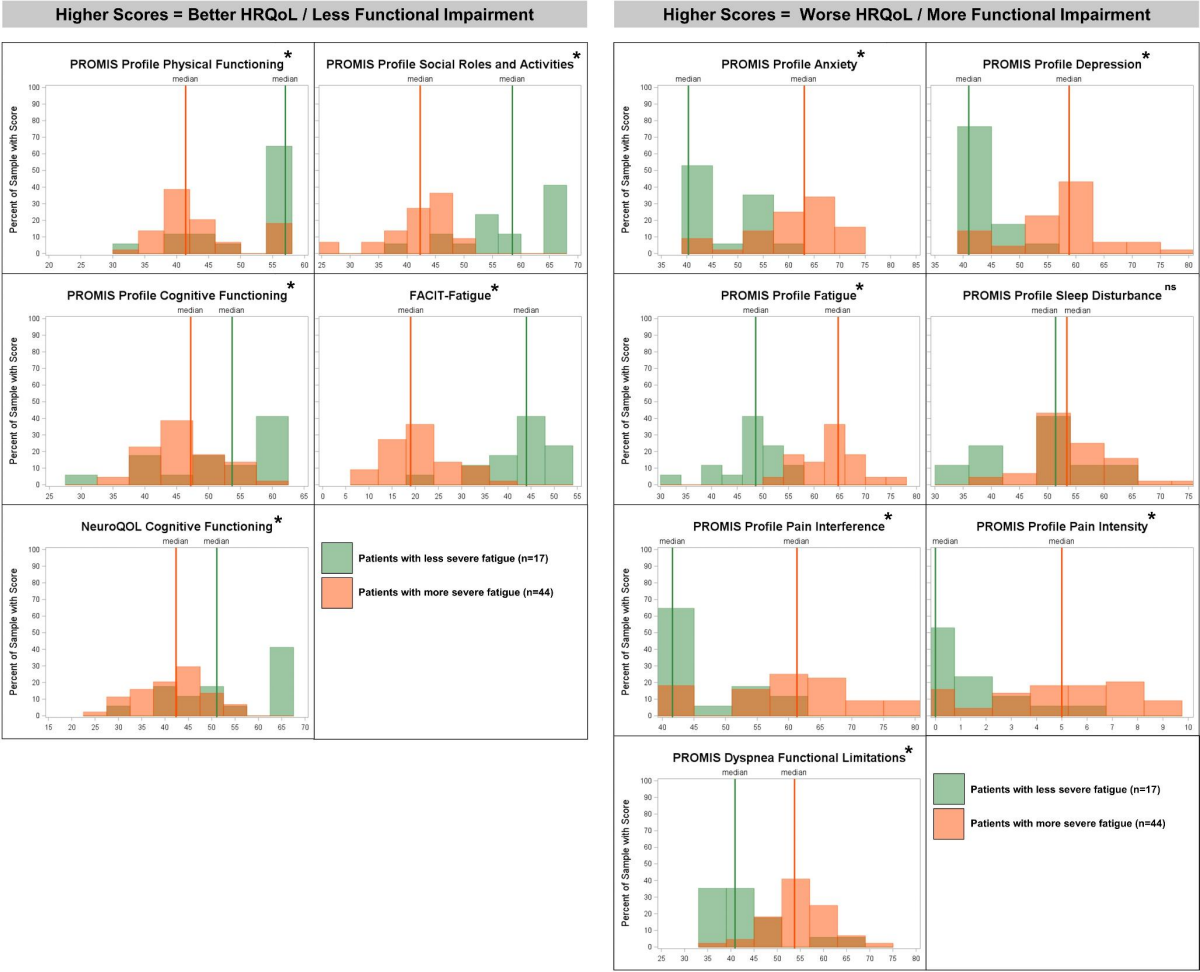
Comparison of distribution of HRQoL and symptom-specific functional impairment scores betweengroups of patients with PNH defined by fatigue severity. *Notes* x-axis corresponds to scores for each domain or measure, restricted to the approximate possible score range. Fatigue severity defined by level of fatigue severity experienced in the past 7 days (very mild or mild vs. moderate, severe, or very severe [no patients reported no fatigue]). * = statistically significant difference, *p* ≤ 0.05; ns = not significant (*p* > 0.05) as evaluated by independent samples *t*-tests or Mann-Whitney tests. *Abbreviations* FACIT, Functional Assessment of Chronic Illness Therapy; HRQoL, health-related quality of life; PNH, paroxysmal nocturnal hemoglobinuria; PROMIS, Patient-Reported Outcomes Measurement Information System; QOL, quality of life

Among patients with PNH, statistically significant relationships were observed between fatigue severity and all domains of HRQoL/symptom-specific functional impairment except sleep disturbance (Table 5; Fig. 3). Patients with PNH who experienced more severe fatigue had worse HRQoL and greater functional impairment than patients with less severe fatigue (corrected *p* < 0.05 for all except the PROMIS Profile Sleep Disturbance scale). The overall pattern of results was maintained when patients with moderate fatigue were grouped with those with very mild or mild fatigue (data not shown).

Fatigue related to PNH was a main theme in all interviews (*n* = 25; 100%), during which participants provided further insights on the specific ways fatigue has impacted their lives. While the FACIT-Fatigue captured patients’ experiences of fatigue over the past 7 days, the interviews allowed for additional insight into fluctuations in and the unpredictability of this experience. For some, fatigue was a daily occurrence, while others experienced it intermittently, ranging from a few times per week to 1–2 times per month to the week before an infusion. One individual described the feeling of fatigue as: “[I]t’s not that you’re sleepy… you just don’t have any energy” (ID 0026, female, age 56), while another described it as feeling “sluggish” (ID 0001, male, age 37). Others described their fatigue as persistent and debilitating (See Table 6 for additional quotes describing fatigue and fatigue-related impairments).

**Table 5:** Comparison of HRQoL and symptom-specific functional impairment between groups of patients with PNH defined by fatigue severity

	Fatigue severity							
	None, Very mild, Mild			Moderate, Severe, Very severe				
Instrument	*n*	Mean (SD)	Median (IQR)	*n*	Mean	Median (IQR)	*P*	*P*_BH_^2^
**PROMIS** ^®^ **29 + 2 Profile v2.1**								
Physical functioning ^↑^	17	51.75 (7.88)	57.00 (45.10, 57.00)	44	43.76 (7.18)	41.40 (39.10, 46.75)	< 0.001	< 0.001
Anxiety	17	46.22 (6.88)	40.30 (40.30, 51.40)	44	60.68 (8.72)	63.05 (56.75, 65.40)	< 0.001	< 0.001
Depression	17	43.16 (4.18)	41.00 (41.00, 41.00)	44	57.43 (8.71)	58.80 (55.10, 62.20)	< 0.001	< 0.001
Fatigue	17	47.29 (5.77)	48.60 (46.00, 50.60)	44	63.07 (5.53)	64.70 (58.55, 66.60)	< 0.001	< 0.001
Sleep disturbance	17	48.46 (9.76)	51.40 (41.20, 53.00)	44	54.24 (6.56)	53.40 (51.10, 56.80)	0.062	0.062
Ability to participate in social roles and activities ^↑^	17	56.65 (8.29)	58.50 (53.60, 64.20)	44	41.13 (5.40)	42.30 (37.20, 44.20)	< 0.001	< 0.001
Pain interference	17	46.55 (7.36)	41.60 (41.60, 53.70)	44	59.88 (10.46)	61.30 (55.65, 67.35)	< 0.001	< 0.001
Cognitive functioning ^↑^	17	51.73 (10.17)	53.70 (43.50, 61.20)	44	46.23 (6.20)	47.20 (41.00, 50.00)	0.022	0.024
Pain intensity	17	1.29 (1.79)	0.00 (0.00, 2.00)	44	4.68 (2.87)	5.00 (3.00, 7.00)	< 0.001	< 0.001
**FACIT-Fatigue**								
Total score ^↑^	17	42.18 (7.45)	44.00 (40.00, 47.00)	44	20.32 (7.34)	19.00 (15.50, 24.00)	< 0.001	< 0.001
**Neuro-QOL Item Bank v2.0 – Cognitive Function – SF**								
Total score ^↑^	17	52.63 (11.11)	51.10 (44.20, 64.20)	44	41.37 (6.87)	42.35 (37.25, 46.20)	< 0.001	< 0.001
**PROMIS Item Bank v1.0 – Dyspnea Functional Limitations – SF**								
Total Score	17	42.92 (9.34)	40.90 (34.80, 45.00)	44	54.33 (7.11)	53.75 (51.05, 60.00)	< 0.001	< 0.001

^↑^ Indicates that higher scores are better; for all other domains, higher scores are worse

*Note* All differences were tested using the Mann-Whitney test for non-normally distributed variables, except for fatigue, which was tested with a *t*-test. If fatigue was evaluated with a Mann-Whitney test, the difference is still significant (*p* < 0.001)

*Abbreviations* BH, Benjamini-Hochberg (correction for multiple comparisons); IQR, interquartile range; PROMIS, Patient-Reported Outcomes Measurement Information System; PNH, paroxysmal nocturnal hemoglobinuria; SD, standard deviation; SF, short form

As noted above, results of the quantitative analyses demonstrated an association between fatigue and functional impairment. For example, 20 (90.9%) of the 22 interview participants who noted PNH impacted their physical functioning attributed these impacts to fatigue. Impacts specifically related to fatigue included the ability to bathe, complete household chores, run errands, and take care of their family, “…it’s not that I’m lazy. There’s a—you know, a lot of people confuse being tired with being lazy. It’s not even that. It’s just I don’t have the energy for washing dishes, doing laundry” (ID 0004, female, age 46).

Fatigue-related impairments on social functioning did not differ greatly from those related to PNH in general. Interview participants (*n* = 15/22 who reported impacts on social functioning; 68.1%) noted their fatigue meant they were hesitant to make plans in advance and/or often found they needed to reschedule or even cancel plans altogether; those who followed through with plans found it exacerbated their fatigue. Five patients (33.3%) specifically described the effects of fatigue on dating relationships or an existing relationship with a partner or spouse, including the need to educate their partner, sexual difficulties, dealing with heightened emotions, cancelling dates, and spending less time together due to the need to rest.

Eight of the participants who reported work-related impacts (*n* = 8/18; 44.4%) attributed these impacts to their fatigue. Further, fatigue contributed to absenteeism for some of these participants (*n* = 3/8; 37.5%), as they found they needed to limit the number of hours they worked. Productivity was also affected; as one participant described, “[S]ometimes getting my work done is a task. It’s hard because I’m already coming to work feeling tired…whatever it is I need to accomplish for the day can be a challenge for me” (ID 0004, female, age 46). One participant explained that because of their fatigue, “it’s just difficult to commit to [a full-time position]… it’s a heavy, heavy burden” (ID 0005, female, age 28).

**Table 6:** Participant quotes describing fatigue-related functional impairment

…housework is almost a no go for me because it’s just more than—more than I can handle. But some days, I get up and I sit on the side of the bed to get up and get ready, and it takes me a while. It’s a process. I can do a little bit at a time… I’ve had to switch from showers to tub baths because I can’t stand up that long… I just can’t go and do grocery shopping, because I can’t walk that long, even though I’m pushing a buggy…it’s just, day-to-day things that you just can’t do because [fatigue] gets in the way. (*ID 0002, female, age 64*)
[S]ocial life with PNH is difficult…dealing with say for instance, family and friends, and having to cancel plans… A lot of people view me as um—almost flaky ‘cause I’ll—I’ll back out of things, but it’s not because I’m being flaky and—and rude, it’s because I’m having symptoms or— sometimes I use—use all my energy doing something else during the day. *(ID 0005*,* female*,* age 28)*
…[S]ome of the things you may want to do…you can’t plan it, you can’t say, “Oh, I know I’m gonna be tired next Tuesday.” Right? You’re like, “Oh, today’s my grandson’s baseball game and I just don’t have the energy to go. *(ID 0015*,* female*,* age 60)*

## Discussion

Results of this real-world, mixed-methods, observational study among patients with PNH show that deficits in HRQoL and functional limitations due to symptoms remain despite receiving treatment with parenterally administered complement inhibitors. Specifically, patients currently treated for PNH with parenterally administered complement inhibitors reported worse HRQoL and greater functional impairment due to fatigue, dyspnea, and cognitive issues than age- and sex-matched adults from the general population. Interviews highlighted the types of everyday activities and life experiences that are affected by PNH, ranging from chores, bathing, and dressing to long-term impacts on career and relationships, while also demonstrating that patients attribute many of these impacts specifically to their fatigue.

This study contributes to a growing body of literature relating to the patient experience with PNH, providing new evidence that emphasizes the unmet needs of patients despite receipt of treatment. Findings from this study are contrary to findings from a study that reported that patients treated for PNH experience better HRQoL and fewer symptoms than the general population [23]. There are several notable factors that could contribute to the observed differences in findings. The previous study included patients who were enrolled in clinical trials of eculizumab and ravulizumab and examined HRQoL after 6 months of treatment comparing scores to pre-existing reference values from the general population. The current study, however, included patients receiving treatment in a real-world setting, excluding those who had participated in a clinical trial for PNH in the past 6 months. Patients had been on their current treatment for an average of 3.2 years; their HRQoL was compared to a general population sample that was recruited specifically for this study to ensure matching on select relevant demographic criteria. These factors – the carefully controlled clinical trial setting, a shorter duration of treatment, and differences in the composition of the comparator group – could all contribute to the differences in findings. Authors of the previous study suggest that treatment helped patients adapt to their condition and recalibrate the way in which they conceptualize the experiences and impacts of their disease. While this response shift may have resulted in reports of *better* HRQoL and *fewer* symptoms than the general population among clinical trial participants over a 6-month period of treatment, results of the current study, though cross-sectional in nature, suggest that this effect may not last in a long-term or real-world setting. These findings, which corroborate the pattern of findings from other real-world studies of treated patients with PNH [8, 24, 25], are particularly important because they demonstrate the need for continued monitoring of patient well-being, implementation of supportive care, or alternative treatment options in an attempt to mitigate the substantial remaining disease burden that exists despite treatment, even if initial increases in well-being and reduction of symptoms are observed when treatment is initiated.

While a critical result of this study was to identify that, despite treatment, patients with PNH still experience unresolved symptoms and deficits in HRQoL, it was equally important to explain and contextualize these experiences in more detail than has been offered by previous research. The survey data show the magnitude of the impact patients experience on various aspects of HRQoL, but the qualitative data extend these findings by also demonstrating how common these experiences are. When both survey and interview data are interpreted together, it becomes clear that the impairments observed, relative to the general population, are likely not driven by a small subset of the patient sample but are a more pervasive experience. The results of this study also provide new information on the interconnected nature of HRQoL, functional impairment, and symptom experiences. For example, while the prevalence of fatigue among patients with PNH has been well documented [6, 7, 24, 26]; the current study extends this work by demonstrating a quantitative association between fatigue and HRQoL and providing additional context from patients that confirms that their experiences of fatigue and HRQoL/functional impairment are strongly intertwined, with some patients directly attributing deficits in physical functioning, social functioning, and employment to their fatigue. As such, the associations identified through quantitative analysis of the survey data are not subtle signals teased out only through a specific analytic approach; they are true reflections of the patients’ experiences, on which they elaborated in depth when provided the opportunity.

As with any study, results should be interpreted within the context of the study’s limitations. Selection bias could influence the composition of both the PNH and general population study samples. For example, patients who experienced greater symptom severity or functional limitations may have been more actively involved in patient advocacy groups or more likely to participate in a study that allowed them to describe their experiences than other patients. The online survey captured HRQoL and symptom-specific functional impairment as they were experienced in the 7 days prior to the survey. Thus, these data were captured at a very specific point in time, which may not be representative of patients’ experiences over time, especially if symptoms fluctuate according to their treatment cycle. The qualitative interviews, however, did not impose similar constraints related to recall period, allowing patients to provide a broader accounting than could be provided through the online survey. The study was descriptive and cross-sectional in nature. Neither causal links nor changes over time could be evaluated. Fitting with the study objectives, analyses were descriptive and did not evaluate how other variables might contribute to the observed pattern of results. Finally, information on comorbidities that could also impact the HRQoL of the study sample (including participants from the general population) was not collected.

Despite these limitations, the study also included several notable strengths. As evidenced by the analysis results and associated interpretation, the mixed-methods design, incorporating both survey and interview-based data, allowed for a more comprehensive exploration of the patient experience. Inclusion of a patient panel to review the study materials prior to data collection helped ensure that both the survey content and interview guide targeted concepts that were relevant to patients with PNH and included language that would be appropriate and understandable. Collecting data from a study-specific, general population sample, rather than reliance on existing benchmark scores - whose composition may differ considerably from the patient sample - provides for a better between-group comparison and ability to accurately detect true differences between the samples.

The findings from this study call for additional attention to the burden of PNH experienced by patients despite treatment with parenterally administered complement inhibitors. Our study illustrates the importance of considering HRQoL and symptom-specific functional impairment, along with clinical endpoints, when identifying and evaluating optimal treatment plans. Such evaluation should occur throughout the course of the disease, even among patients who are not newly diagnosed or who have not recently switched treatments. Continued investigation of novel treatment options is also essential, especially to identify those that reduce or eliminate breakthrough symptoms such as fatigue, while also minimizing the burden of treatment administration. Availability of new treatments, including those with different half-lives or methods of administration, have the potential to improve patients’ HRQoL in meaningful ways. Future research should focus on exploring interventions that could improve patient outcomes, particularly to better understand how reductions in fatigue may contribute to better HRQoL.

## Data Availability

Raw data are not publicly available due to confidentiality and consent limitations.
